# Genetic Interactions Between *Aspergillus fumigatus* Basic Leucine Zipper (bZIP) Transcription Factors AtfA, AtfB, AtfC, and AtfD

**DOI:** 10.3389/ffunb.2021.632048

**Published:** 2021-02-11

**Authors:** Lilian Pereira Silva, Maria Augusta Crivelente Horta, Gustavo Henrique Goldman

**Affiliations:** Faculdade de Ciências Farmacêuticas de Ribeirão Preto, Universidade de São Paulo, São Paulo, Brazil

**Keywords:** *Aspergillus fumigatus*, transcription factors, mitogen-activated protein kinase, high osmolarity glycerol, cell wall integrity

## Abstract

*Aspergillus fumigatus* is an opportunistic fungus, capable of causing Invasive Aspergillosis in immunocompromised patients, recently transplanted or undergoing chemotherapy. In the present work, we continued the investigation on *A. fumigatus* AtfA-D transcription factors (TFs) characterizing possible genetic and physical interactions between them after normal growth and stressing conditions. We constructed double null mutants for all the possible combinations of Δ*atfA-, -B, -C*, and *-D*, and look into their susceptibility to different stressing conditions. Our results indicate complex genetic interactions among these TFs that could impact the response to different kinds of stressful conditions. AtfA-D interactions also affect the *A. fumigatus* virulence in *Galleria mellonella*. AtfA:GFP is ~97% located in the nucleus while about 20–30% of AtfB, -C, and -D:GFP locate into the nucleus in the absence of any stress. Under stressing conditions, AtfB, -C, and -D:GFP translocate to the nucleus about 60–80% upon the addition of sorbitol or H_2_O_2._ These four TFs are also interacting physically forming all the possible combinations of heterodimers. We also identified that AtfA-D physically interact with the MAPK SakA in the absence of any stress and upon osmotic and cell wall stresses. They are involved in the accumulation of trehalose, glycogen and metabolic assimilation of different carbon sources.

## Introduction

*Aspergillus fumigatus*, is a filamentous saprophytic fungus, which is commonly found in the environment (Prasad et al., 2016). This fungus produces conidia, which facilitates its dispersion in the air, and due to its small size (2–3 μm) and its abundance in the environment, humans inhale daily hundreds of these conidia, which can lodge in their lower respiratory tract (Tischler and Hohl, 2019) and cause the disease called Invasive Pulmonary Aspergillosis (IA) (Prasad et al., 2016). The mortality rate of infection caused by *A. fumigatus* can reach 85% in immunocompromised individuals or those who have undergone transplantation and/or chemotherapy procedures. In these patients, the lines of defense, such as coordinated action of the respiratory epithelium, resident macrophages in the lungs, neutrophils and monocytes, recruited by the host to eliminate conidia do not work efficiently (van de Veerdonk et al., 2017; Tischler and Hohl, 2019). The virulence of the pathogen is multifactorial, where several phenotypes can influence the establishment of the disease. It is known that different signaling pathways used by *A. fumigatus* are responsible for modulating the biochemical machinery of the pathogen, thus favoring rapid adaptation to different environmental stresses and the host immune system (Silva et al., 2017). The highly conserved mitogen-activated protein kinase (MAPK) is one of these signaling pathways (Pearson et al., 2001; Rispail et al., 2009; Day and Quinn, 2019). MAPKs are important for relaying, integrating, and amplifying intracellular signals involved in many cellular processes (Pearson et al., 2001; Rispail et al., 2009). *A. fumigatus* has four MAPK cascades (i) MpkA, a central regulator of the cell wall integrity pathway (CWI) (Valiante et al., 2015); (ii) MpkB, involved in the regulation of the production of secondary metabolites, in particular DHN-melanin (Manfiolli et al., 2019; da Costa Filho et al., 2020); and (iii) MpkC and SakA, orthologs of *S. cerevisiae* Hog1p, the main regulator of the HOG-MAPK pathway (Rispail et al., 2009; Silva et al., 2017).

The central signal transduction pathway operating during hyperosmotic and oxidative stresses is the High Osmolarity Glycerol (HOG) MAPK cascade (Martínez-Montañés et al., 2010; de Nadal and Posas, 2015). In yeast, the HOG pathway has signaling branches controlled by the osmosensors Sln1p and Sho1p, with independently function (Hohmann, 2002). Under stressful conditions Sln1p phosphorylates Ypd1p which transfers its phosphate to the Ssk1p regulator, initiating the kinases phosphorylation of the HOG pathway. Another branch comprises the osmosensor Sho1p, putative osmosensors Hkr1p, Msb2p, and the membrane anchor protein Opy2p, responsible for initiating the process of activating the cascade of kinases Ste11p, Pbs2p, and Hog1p (Hohmann, 2002). Recently, we have shown that some elements of these pathways are conserved in *A. fumigatus* activating not only SakA homolog but also MpkA, a MAPK from the CWI (Manfiolli et al., 2019; Silva et al., 2020). Upon different kinds of stress *A. nidulans* SakA and *A. fumigatus* SakA and MpkC are translocated to the nucleus (Lara-Rojas et al., 2011; Bruder Nascimento et al., 2016). *A. nidulans* SakA accumulates in the nucleus and physically interacts with the basic leucine zipper transcription factor (TF b-ZIP) AtfA^Atf1p^, which activates catalase genes during oxidative stress (Lara-Rojas et al., 2011). This TF, the mammalian ATF2 homolog, binds to CRE regions [T [G/T] ACGT [C/A] A] of target genes in response to stressful conditions (Hagiwara et al., 2008; Sakamoto et al., 2009). In *A. fumigatus* AtfA homolog was first described as dependent on the stress-activated kinase SakA and the cyclic AMP-protein kinase A (cAMP-PKA) pathways and important for the expression of DprA dehydrin, a protein important to cope with oxidative stress conditions and phagocytic killing (Hoi et al., 2011). *A. fumigatus atfA* null mutant was shown to be important for long-term viability of conidia, with significant sensitivity to high temperature and oxidative stress, and reduced conidial trehalose content (Hagiwara et al., 2014). Transcriptomic analysis comparing hyphae, resting conidia, and 1-h grown conidia in *A. fumigatus, A. niger*, and *A. oryzae* showed that AtfA is important for the regulation of conidial stress-related and germination genes, suggesting a major role for AtfA in *Aspergillus* conidial dormancy (Hagiwara et al., 2016). These authors also identified three putative AtfA homologs, AtfB, -C, and -D that have a relatively conserved bZIP-type DNA-binding domain (Hagiwara et al., 2014). We have characterized null mutants for all these four *A. fumigatus* b-ZIP TFs. These four TFs have their expression dependent on Δ*sakA* and Δ*mpkC* Δ*sakA* post-osmotic stress, and displayed several phenotypes related to osmotic, oxidative, and cell wall stresses (Silva et al., 2017). The Δ*atfA* and Δ*atfB* were shown to be avirulent and to have attenuated virulence, respectively, in both *Galleria mellonella* and a neutropenic murine model of invasive pulmonary aspergillosis (Silva et al., 2017).

TFs have the potential to elucidate virulence mechanisms and provide information that can help us highlight new targets for antifungal drugs. TFs, abundantly regulate several important genes for a given biological response (Bultman et al., 2017). In addition, b-ZIP TFs have the ability to form homo- or heterodimers, a paradigm that provides combinatorial control of gene expression in eukaryotes, increasing the complexity of the genetic regulatory network and allowing the emergence of new control circuits (Klemm et al., 1998; Pogenberg et al., 2014). In the present work, we continued the investigation on *A. fumigatus* AtfA-D TFs characterizing possible genetic and physical interactions between them after normal growth and stressing conditions. Here, we showed that these four TFs are not only interacting at genetic level but also physically forming all the possible combinations of heterodimers. We also identified that AtfA-D physically interact with the MAPK SakA in the absence of any stress and upon osmotic and cell wall stresses.

## Materials and Methods

### Strains and Culture Media

The *A. fumigatus* strains used during the study are listed in the Supplementary Table 1. The culture used were mainly complete and/or minimal media. The complete medium consists of, YAG (2% w/v dextrose, 0.5% w/v yeast extract, 0.1% v/v element trace solution, 2% w/v agar), YUU (Medium YAG plus uridine and uracil 1.2 g/L), and YG or YG + UU media with the same composition, but without agar. The minimum medium (MM) consists of 1% glucose, 0.1 element trace solution % v/v, 20X salt solutions, pH 6.5 with or without the addition of 2% (w/v) agar (Kafer, 1977). The strains were grown at 37°C for 5 days.

### Construction of Mutant Strains

All sequences are in accordance with the AspGD database (http://www.aspgd.org/). The deletion cassette was constructed by *in vivo* recombination in *S. cerevisiae* as described (Colot et al., 2006). About 1.0 kb of the flanking region of the 5′-UTR and 3′-UTR regions of the target ORF regions were selected for primer design. The 5′Fw and 3Rv primers contained a short sequence homologous to plasmid pRS426 and a checkmark pyrG or pPTRA. Both 5′and 3′-UTR fragments were amplified by PCR from *A. fumigatus* genomic DNA (gDNA). The *pyrG* gene placed inside the cassette as a prototrophic marker was amplified from the vector pCDA21. The deletion cassette was generated by transformation into *S. cerevisiae* SC94721, using the lithium acetate method (Schiestl and Gietz, 1989), plus the fragments, together with the plasmid pRS426 digested in two sites with the enzyme *BamHI* and *EcoRI* (New England Biolabs UK). The DNA of the transformants was extracted using the method described (Goldman et al., 2003). The deletion cassette was amplified by PCR from these plasmids using TaKaRa Ex Taq ™ DNA polymerase (Clontech Takara Bio) and used for the transformation of *A. fumigatus*. The same protocol was applied to the construction of the double mutants. However, the check mark used for the gene deletion was amplified from the plasmid pPTRI. Colonies of null mutants were selected by purifying colonies in MM with 1 μg/ml pyriamiamine hydrobromide (Sigma). Southern blot analysis demonstrated that the transformation cassette was homologous. Deletion strains of a single gene were complemented by co-transformation, ~1.0 kb of the 5′ and 3′-UTR plus ORF together with the plasmid pPTRI according to Herrera-Estrella et al. (1990) with modifications. Complements were selected by purifying colonies in MM with 1 ug/ml of piritiamine hydrobromide.

The homologous and/or heterologous recombination to gene replacement was confirmed by PCR and Southern blot. The absence of mRNA from the target genes was verified by qPCR. All primers used for the constructions are listed in Supplementary Table 2.

### Characterization of Mutant Null Strains

The characterization of sensitivity and/or resistance was evaluated by radial growth comparing mutant and wild strains. Five microliter of a 2 × 10^7^ suspension were dropped into MM with or without stressors, the plates were cultured for 5 days at 37°C. After that period, the radial diameter was measured and the values were used for statistical analysis. The different stressful conditions were ions a with the addition of calcofluor white (CFW), Congo Red (CR), menadione, sorbitol, and caspofungin.

### Growth of Strains in Alternative Carbon Source

To assess growth in different carbon sources, we use modified MM. Sodium acetate (1%) or ethanol (1%) was added to the MM instead of glucose (1%) (Kafer, 1977). The strains were grown for 5 days at 37°C.

### Statistical Analysis

Data were analyzed using (Prism, GraphPad) “Two-way ANOVA” followed by “Bonferroni post-tests.” Significance levels were ^*^p < 0.1, ^**^p < 0.01, and ^***^p < 0.001. All the strains were compared with the single mutants and the Wild-type.

### Protein Extraction and Western Blot

The strains were inoculated (1 × 10^7^ conidia) in 250-ml erlenmeyer's flasks with 50 ml of YG medium and grown in a rotatory shaker (200 rpm) at 37°C for 16 h before being exposed to different stressing agents, such as Congo Red, sorbitol. The total protein extracts were obtained through mycelia ground with The macerated mycelia were resuspended (500 mg) in 1 ml of B250 lysis buffer (250 mM NaCl, 100 mM Tris-HCl pH 7.5, 10% glycerol, 1 Mm EDTA and 0.1% NP-40) supplemented with 1.5 ml/L of 1M DTT, 100 mL of complete protease inhibitor Cocktail without EDTA (Roche), 3 ml/L of 0.5M Benzamidine, 10 ml/L of 100X phosphatase inhibitors (1M NaF, 5M sodium orthovanadate, 8M glycerol phosphate) and 10 mL/L of 100 mM PMSF. The samples were centrifuged at 13,000 g for 10 min at 4°C. The supernatants were removed and quantified by Bradford assay (BioRad). Western blots were performed as previously described (de Assis et al., 2018).

### GFP-Trap and Anti-HA (Co-IP)

To perform the co-immunoprecipitation (co-IP) assays, AtfA-D:GFP strains had SakA marked with 3xHA at the C-terminus (SakA:3xHA). The strains were grown for 16 h in liquid YG and were further exposed to two conditions, these being, sorbitol 1.2 M (10 min) and CR 300 ug/ml (10 min). The co-IP experiments were carried out with GFP-Trap (Chromoteck) according to Manfiolli et al. (2017). To perform co-IP tests, the mycelia were frozen with liquid nitrogen and macerated with the aid of a crucible and pistil. The samples were normalized and the same amount of protein was added to 20 μL of GFP-trap, the resin was washed three times with resuspension buffer before incubation. The protein extracts and the resin were then incubated with shaking at 4°C for 4 h. After incubation, the resin was washed three times in B250 buffer. To release proteins from the resin, the samples were incubated with sample buffer and boiled at 98°C for 5 min. The proteins were transferred from a 10% SDS-PAGE gel to a nitrocellulose membrane for a Western blot assay using a Trans-Blot turbo transfer system (Bio-Rad). GFP-labeled AtfA-D were detected using a rabbit anti-GFP antibody (Cell Signaling Technology) at a dilution of 1:1,000. For the detection of HA-labeled proteins, a mouse anti-HA monoclonal antibody (Sigma) was used in the 1:2,000 dilution. Both primary antibodies were identified with the conjugated anti-mouse IgG antibody (Cell Signaling Technology) used at 1:1,000 dilution as a secondary antibody.

### Measurement of Glucose in the Supernatant

The strains were inoculated (1 × 10^7^ conidia) in 250 ml erlenmeyer's flasks with 50 ml of YG medium and grown in a rotatory shaker (200 rpm) at 37°C for 24 h. After that period, the mycelia were then transferred to minimum medium plus 1% glucose as a carbon source for another 24 h. The amount of free glucose remaining in the medium was measured using the GOD-PAP Glucose Liquid Stable Monoreagent kit (Laboratories LaborLab Ltd. Guarulhos, São Paulo, Brazil), according to the manufacturer's instructions. Glucose uptake was calculated by determining the difference in glucose present in the initial medium and after 24 h of incubation.

### Mycelia Dry Weight

The strains were inoculated (1 × 10^7^ conidia) in 250 ml erlenmeyer's flasks with 50 ml of YG medium and grown in a rotatory shaker (200 rpm) at 37°C for 24 h. After that period, the mycelia were transferred to minimum medium plus 1% glucose as a carbon source for 24 h. Then the mycelia were filtered and taken to the lyophilizate for 48 h. The dry weight of each strain was compared to the dry weight of the wild type mycelium to normalization.

### Intracellular Glycogen and Trehalose Concentrations

Intracellular glycogen and trehalose levels were measured in mycelia grown 24 h in MM supplemented with glucose as a carbon source as previously described (Manfiolli et al., 2019).

### Virulence Analysis in *Galleria mellonella* Models

*Galleria mellonella* larvae were obtained by reproducing adult larvae (Fuchs et al., 2010). The larvae used for the infection were in the last larval stage of development (sixth week). All selected larvae weighed ~300 mg, and were restricted to food for 24 h before the experiment. The fresh conidia of each *A. fumigatus* strain were counted using a hemocytometer. The initial concentration of conidial suspensions for infections were 2 × 10^8^ conidia/ml, total of 5 μl (1 × 10^6^ conidia/larvae) of each suspension was inoculated by larva. The control group was composed of larvae whose 5 μl of PBS were injected to observe death from physical trauma. The inoculum was carried out with the Hamilton syringe (7000.5 KH) through the last left ear. After infection, the larvae were kept with food restrictions, at 37°C in Petri dishes in the dark, and scored daily. The larvae were considered dead due to lack of movement in response to touch. The viability of the inoculum administered was determined by serial dilution of the conidia in YAG medium and incubating the plates at 37°C for 72 h.

## Results

### Double Mutants for the TFs AtfA-D Display Increased Sensitivity to Osmotic, Oxidative, and Cell Wall Stresses in *A. fumigatus*

**Figure 1:**
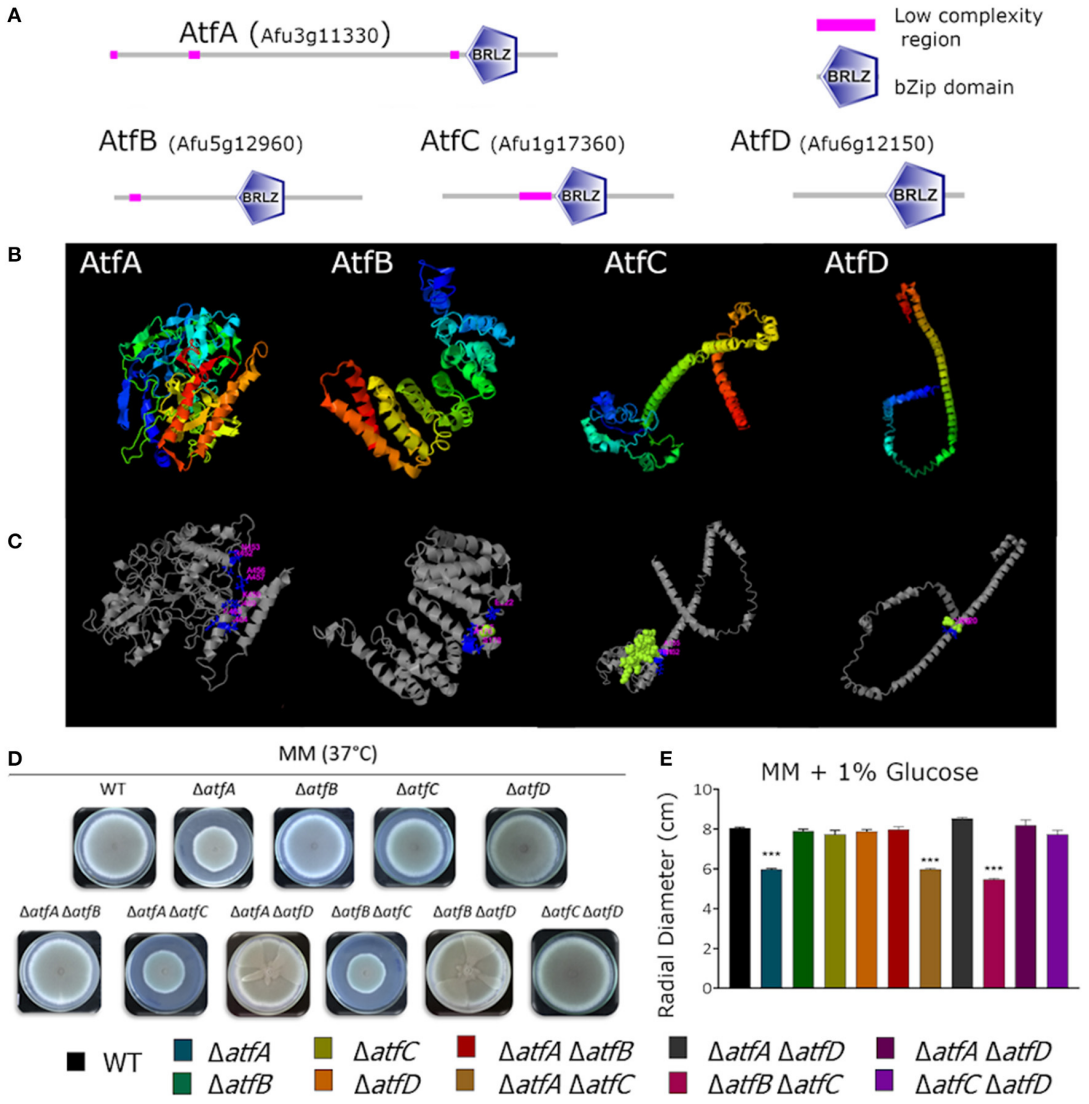
Predicted AtfA-D protein structures and comparative phenotypic growth of the wild-type and Δ*atfA-D* mutants. **(A)** *A. fumigatus* protein structure of AtfA-D family. Protein sequence AtfA (Afu3g11330), length 555 aminoacids (aa), conserved bZip domain sequence between aa 441 and 505; AtfB (Afu5g12960), length 328 aa, conserved bZip domain sequence between aa 161 and 225; AtfC (Afu1g17360), length 286 aa, conserved bZip domain sequence between aa 139 and 203; AtfD (Afu6g12150), length 185 aa, conserved bZip domain sequence between aa 100 and 164aa. **(B)** Modeling prediction of protein structure C-score/TM-score: AtfA −0.55/0.64 ± 0.13, AtfB −2.57/0.42 ± 0.14, AtfC −2.78/0.40 ± 0.13, AtfD −2.10/0.46 ± 0.15. **(C)** Deduction of binding site position by structural comparison PDB Hit/C-score: AtfA 2h7hB/0.11, AtfB 4evpA/0.11, AtfC 3j47U/0.15, AtfD 3hd7A/0.18. **(D,E)**. Growth phenotypes of the wild-type, Δ*atfA* Δ*atfB*, Δ*atfA* Δ*atfC*, Δ*atfA* Δ*atfD*, Δ*atfB* Δ*atfC*, Δ*atfB* Δ*atfD*, and Δ*atfC* Δ*atfD* strains. The strains were grown for 5 days at 37°C on MM. The data were analyzed using (Prism, GraphPad) “Two-way ANOVA” followed by “Bonferroni post-tests.” The levels of significance were of ***p < 0.001 compared to the wild-type strain.

In a previous publication by our research group (Silva et al., 2017), we showed that the genes encoding the TFs AtfA-D have their mRNA accumulation dependent on MAP kinases SakA and MpkC and possibly function downstream the HOG pathway regulating genes involved in the adaptation of *A. fumigatus* to osmotic stress. These four TFs belong to a small bZIP family whose proteins are between 185 and 555 aa (Figure 1A). Multiple alignment of the bZIP domain of AtfA has about 58–65% identity with AtfB-D. The bZip domains were identified by SMART (http://smart.embl-heidelberg.de) and Figure 1A shows the linear protein structures with the correspondent bZip domain position. Protein modeling predictions based on structural templates allowed to observe the AtfA-D structural configuration (Figure 1B) and the binding site deduced region (Figure 1C). To further characterize these TFs, we constructed double deletions mutants for these four null TF genes, therefore generating six double mutants (Δ*atfA* Δ*atfB*, Δ*atfA* Δ*atfC*, Δ*atfA* Δ*atfD*, Δ*atfB* Δ*atfC*, Δ*atfB* Δ*atfD*, and Δ*atfC* Δ*atfD*) and evaluated the possible genetic interactions among the mutants in the presence of different stressing conditions.

Growth of the double deletion mutants in minimum medium (MM) for 5 days at 37°C provided initial evidence of the differences and synergism between single TF mutants (Figures 1D,E). The mutant Δ*atfA* displayed reduced growth when compared to the wild-type and the other single deletion mutants (Figures 1D,E). Deleting either *atfB* or *atfD* in the Δ*atfA* background restored wild-type phenotype suggesting these two mutations can suppress the Δ*atfA* growth defect (Figures 1D,E); however, deleting *atfC* in the same background mimicked the Δ*atfA* phenotype (Figures 1D,E). Reduced growth was also observed for the Δ*atfB* Δ*atfC* mutant (Figures 1D,E). Interestingly, deleting *atfD* in both Δ*atfA*, and Δ*atfB* backgrounds resulted in sectorized growth, what was not observed for Δ*atfC* Δ*atfD* (Figure 1D). Table 1 shows the different genetic interactions between the mutants.

**Table 1:** Summary of the genetic interactions between *atfA, atfB, atfC*, e *atfD* double mutants grown on different stressing conditions.

Strains/Phenoypes*	**Δ*atfA* Δ*atfB***	**Δ*atfA* Δ*atfC***	**Δ*atfA* Δ*atfD***	**Δ*atfB* Δ*atfC***	**Δ*atfB ΔatfD***	**Δ*atfC* Δ*atfD***
CFW	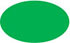	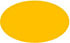	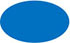	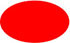	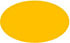	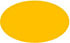
CR	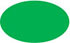	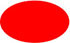	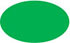	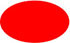	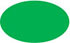	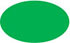
Sorbitol	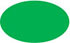	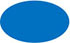	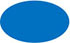	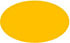	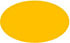	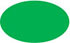
H_2_O_2_	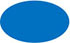	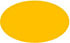	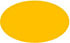	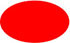	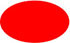	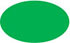
Menadione	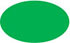	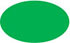	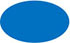	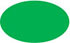	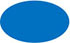	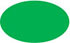
MM+glucose	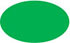	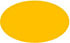	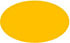	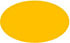	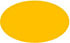	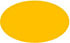
MM+acetate	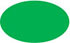	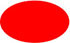	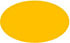	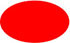	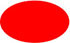	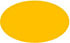
MM+ethanol	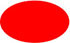	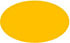	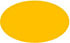	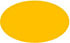	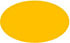	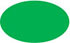
Trehalose	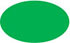	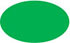	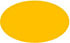	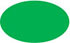	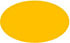	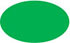
Glycogen	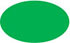	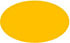	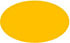	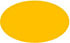	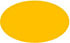	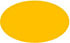
Virulence	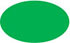	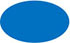	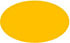	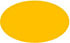	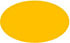	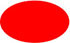

*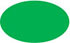 epistatic interaction; 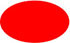 additive interaction; 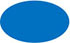 suppression interaction; 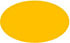 no interaction*.

*MM, Minimal media 1% glucose; CR, Congo Red (50 μg/ml); CFW, Calcofluor White (90 μg/ml); CaCl_2_ (500 mM); Sorbitol 1.2 M; Menadione 0.05 M;MM+glucose 1%; MM+acetate 1%; MM+ethanol 1%; and virulence in Galleria mellonella.

**Figure 2:**
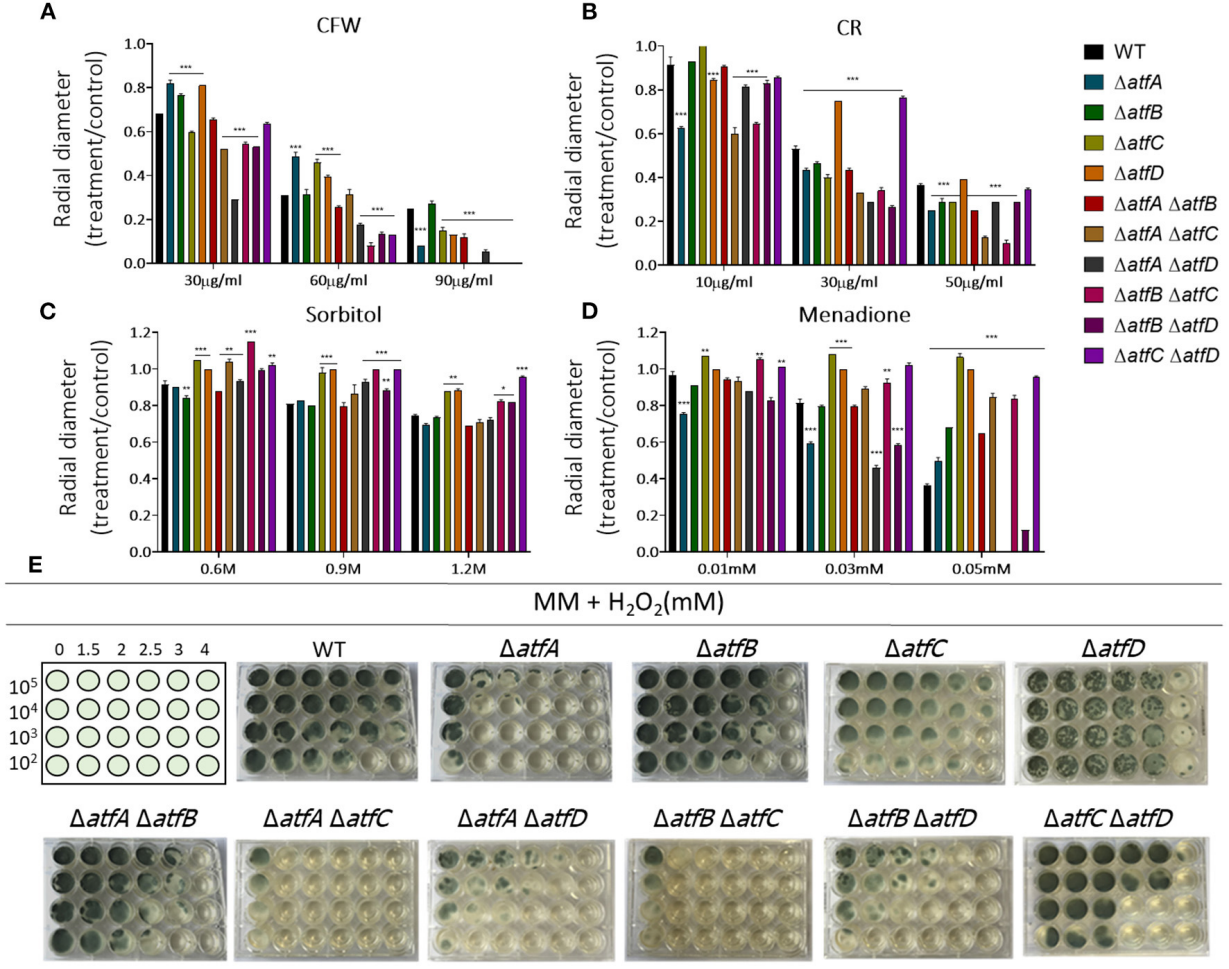
*A. fumigatus* double null mutants indicate genetic interactions among Δ*atfA*, Δ*atfB*, Δ*atfC*, and Δ*atfD*. Growth phenotypes of the wild-type, Δ*atfA* Δ*atfB*, Δ*atfA* Δ*atfC*, Δ*atfA* Δ*atfD*, Δ*atfB* Δ*atfC*, Δ*atfB* Δ*atfD*, and Δ*atfC* Δ*atfD* strains. **(A)** MM+Calcofluor White (CFW). **(B)** MM+Congo Red (CR). **(C)** MM+sorbitol. **(D)** MM+menadione, and had their radial growth quantified. The results are the means of measuring the diameter of 3 colonies for each strain ± standard deviation. The data were analyzed using (Prism, GraphPad) “Two-way ANOVA” followed by “Bonferroni post-tests.” The levels of significance were of *p < 0.1, **p < 0.01, and ***p < 0.001 compared to the wild-type strain. **(E)** Liquid MM+H_2_O_2_. The strains were grown for 72 h at 37°C.

In the presence of CFW, the double Δ*atfA* Δ*atfB* is as sensitive as Δ*atfA* and Δ*atfB* to CFW (90 μg/ml) while Δ*atfA* Δ*atfC* recapitulates Δ*atfA* growth (Figure 2A). The Δ*atfB* Δ*atfC* mutant is more sensitive to CFW (90 μg/ml) than the corresponding single mutants, suggesting additive interaction between these two mutants (Figure 2A, Table 1). Deleting *atfD* in the Δ*atfA* background restored wild-type phenotype suggesting this mutation can suppress the Δ*atfA* growth defect (Figure 2A). The Δ*atfB* Δ*atfD* and Δ*atfC* Δ*atfD* mutants are as sensitive as the single mutants, suggesting the single mutants are not interacting (Figure 2A, Table 1). In the presence of CR (50 μg/ml), all the double mutants behave like the single mutants, except for Δ*atfA* Δ*atfC* and Δ*atfB* Δ*atfC* that are more sensitive than the corresponding single mutants, suggesting additive interaction (Figure 2B, Table 1). The double mutants Δ*atfB* Δ*atfC*, Δ*atfB* Δ*atfD* and Δ*atfC* Δ*atfD* are more resistant to higher osmotic concentrations (1.2 M sorbitol) mimicking the single mutant phenotypes of Δ*atfC* and Δ*atfD* (Figure 2C, Table 1). In contrast, deletion of *atfA* in Δ*atfC* and Δ*atfD* background increase the sensitivity to 1.2 M sorbitol, suggesting a suppression phenotype (Figure 2C, Table 1).

We also investigated the response to two agents that cause oxidative stress, H_2_O_2_ and menadione (Figures 2D,E). Growth in different H_2_O_2_ concentrations showed that Δ*atfA* Δ*atfC* and Δ*atfA* Δ*atfD* recapitulate the Δ*atfA* phenotype, suggesting there are no genetic interactions between these mutants (Figure 2E, Table 1). In contrast deleting *atfB* in the Δ*atfA* background partially suppresses the H_2_O_2_ sensitivity while Δ*atfB* in the Δ*atfC* or Δ*atfD* background increases H_2_O_2_ sensitivity (Figure 2E, Table 1). In the presence of 0.05 mM menadione, the double mutants Δ*atfA* Δ*atfB*, Δ*atfA* Δ*atfC*, Δ*atfB* Δ*atfC*, and Δ*atfA* Δ*atfD* are as sensitive as the corresponding single mutants (Figure 2D, Table 1), In contrast the deletion of *atfD* in the Δ*atfA* or Δ*atfD* background increases the H_2_O_2_ sensitivity (Figure 2E, Table 1).

The results observed here for the single mutants are similar to those reported by Silva et al. (2017). However, the data obtained with the analysis of the double mutants indicate complex genetic interactions among these TFs that could impact the response to different kinds of stressful conditions.

### *A. fumigatus* AtfA-D:GFP Nuclear Localization

**Figure 3:**
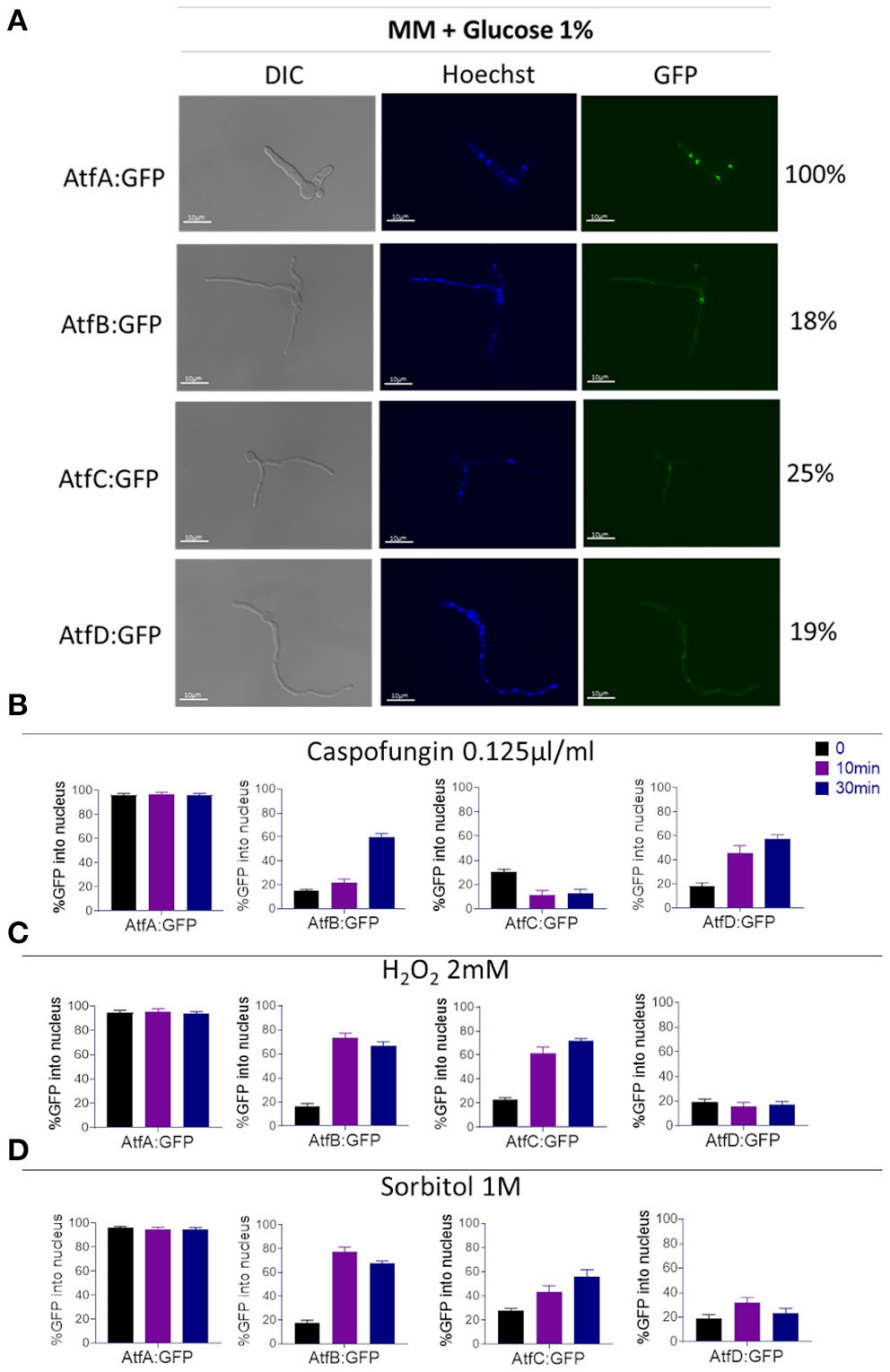
Determination of the AtfA-D:GFP nuclear localization upon different stressing conditions. **(A)** Cellular locatizaton (%) of the *A. fumigatus* AtfA-D:GFP in liquid MM. **(B)** Percentage of cellular localization of AtfA-D:GFP proteins after treatment with caspofungin (0.125 ug/ml). **(C)** Percentage of cellular localization of AtfA-D: GFP proteins after treatment with H_2_O_2_ 2 mM. **(D)** Percentage of cellular localization of AtfA-D: GFP proteins after treatment with sorbitol 1.0 M. The AtfA-D:GFP strains were grown in liquid MM for 16 h at 30°C, after that the treatments with stressors were performed.

Because the cell localization of TFs AtfA-D in *A. fumigatus* has not been previously described we investigate the nuclear localization of these TFs in functional AtfA-D:GPF strains (Figure 3A, Supplementary Figure 1). AtfA:GFP is ~97% located in the nucleus of *A. fumigatus*, independently of exposure or not to stressing conditions (Figures 3A–D). In contrast, about 20–30% of AtfB, -C, and -D:GFP locates into the nucleus (Figures 3A–D). However, under stressing conditions, these TFs tend to translocate to the nucleus. AtfB:GFP translocates to the nucleus about 60–80% upon the addition of sorbitol, H_2_O_2_, or caspofungin (Figures 3A–D). AtfC:GFP translocates about 65–80% upon addition of sorbitol and H_2_O_2_ but only 10% in the presence of caspofungin (Figures 3A–D). Finally, AtfD:GFP translocates 30–60 in the presence of sorbitol and H_2_O_2_, respectively, but 60% in the presence of caspofungin (Figures 3A–D).

These data suggest AtfA-D are possibly important for the transcriptional regulation of genes involved in the response to osmotic, oxidative, and cell wall stresses.

### The TFs AtfA-D Contribute to the Adaptation of *A. fumigatus* to Different Carbon Sources

**Figure 4:**
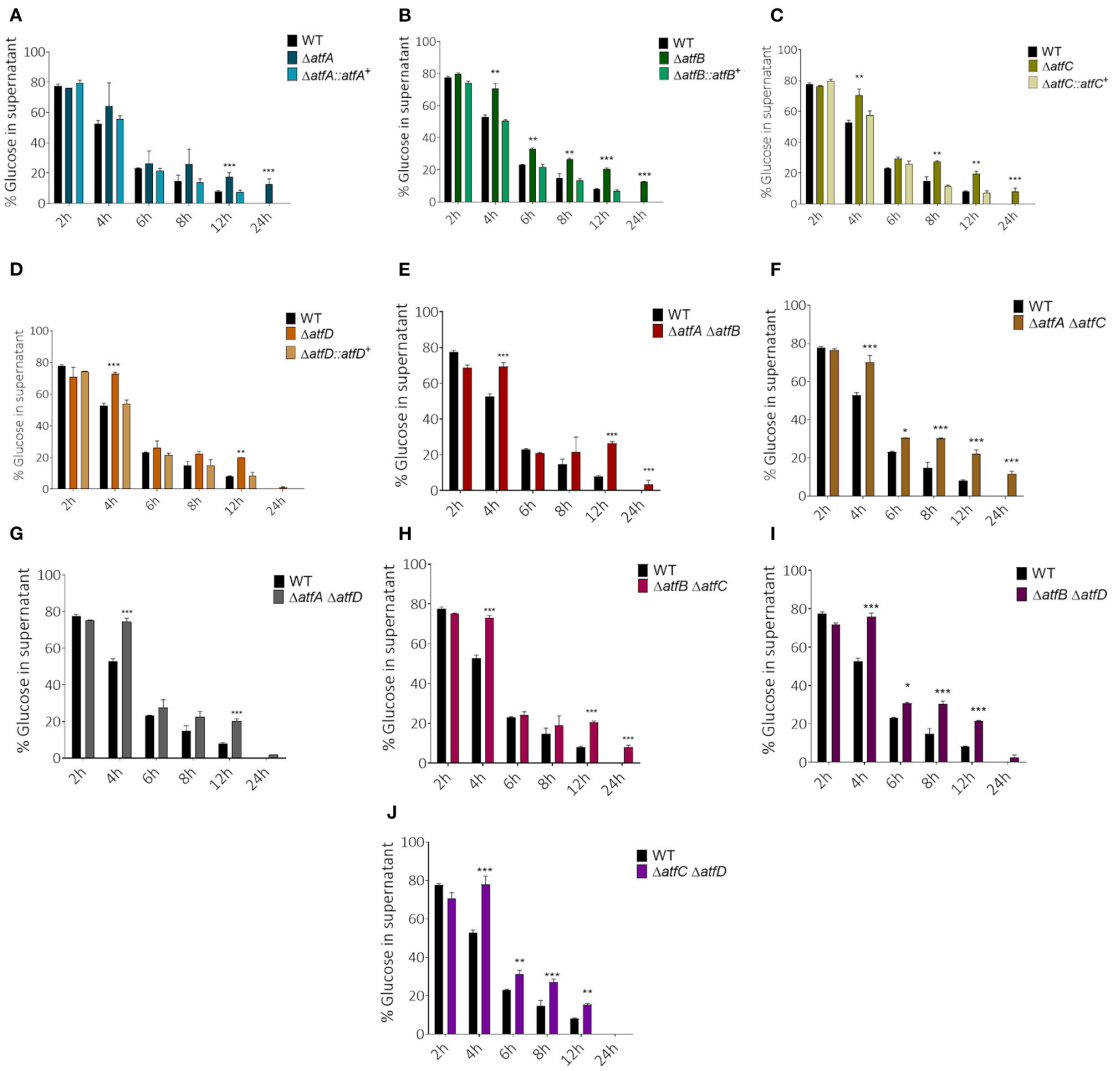
Measurement of glucose concentration in the supernatant. **(A–D)** Simple *atfA-D* mutants and their complemented strains. **(E–J)** Measurement of glucose available in MM after the growth of *atfA-D* double mutants compared with thewild-type strain. Aliquots of the supernatant of three repetitions were collected at different time points (2, 4, 6, 8, 12, and 24 h). The data were analyzed using (Prism, GraphPad) “Two-way ANOVA” followed by “Bonferroni post-tests.” The levels of significance were of **p < 0.01 and ***p < 0.001 compared to the wild-type strain. *p < 0.1.

To verify if AtfA-D could influence carbon source utilization we checked the glucose consumption rate in liquid medium for single and double deletion mutants. Single mutants show slower glucose consumption when compared to the wild-type (Figures 4A–D). While the wild-type and Δ*atfD* exhaust all glucose in 24 h, the Δ*atfA* and Δ*atfB* strains consumed only 80% of glucose and Δ*atfC* consumed 90%. The Δ*atfA* Δ*atfB*, Δ*atfA* Δ*atfC*, Δ*atfB* Δ*atfC* mimicked the phenotypes of either Δ*atfA*, Δ*atfB*, or Δ*atfC* (Figures 4E,F,H, Table 1) while deletion of either *atfA, atfB*, or *atfC* in the Δ*atfD* recapitulates the Δ*atfD* phenotype (Figures 4G,I,J, Table 1). We measure the dry weight of mycelia to evaluate if a reduced glucose consumption would affect the growth of strains (Supplementary Figure 2). The single mutant Δ*atfA* shows a 50% reduction in dry weight when compared to the wild-type (Supplementary Figure 2A), while the double mutants Δ*atfA* Δ*atfC*, Δ*atfA* Δ*atfD*, Δ*atfB* Δ*atfC*, and Δ*atfB* Δ*atfD* show 40–50% dry weight reduction when compared to the wild-type strain (Supplementary Figure 2B).

**Figure 5:**
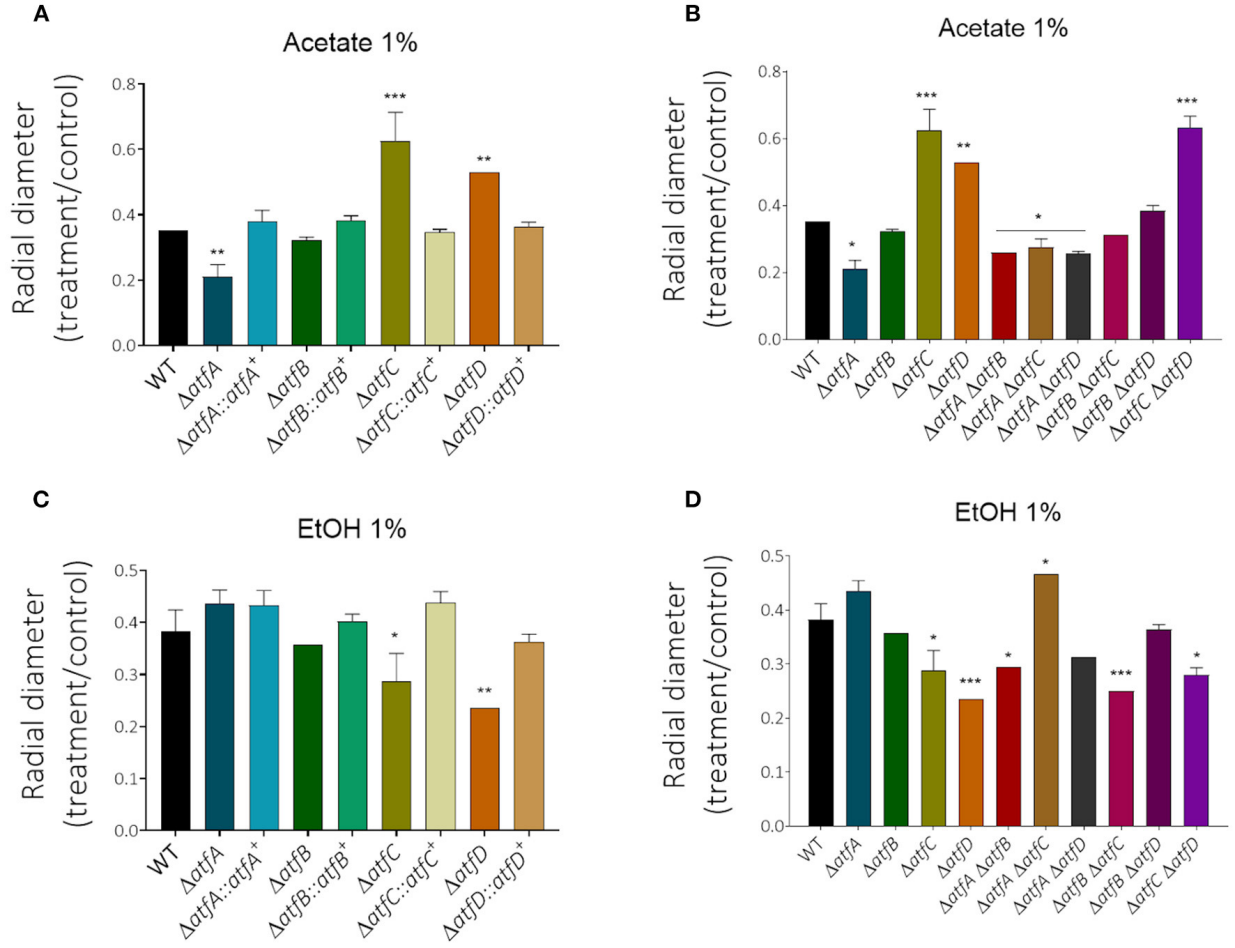
The radial growth of Δ*atfA-D* single and double null mutants on acetate or ethanol as single carbon sources. The wild-type and null mutants were grown for 5 days at 37°C. The results are the means of measuring the diameter of 3 colonies for each strain ± standard deviation. The data were analyzed using (Prism, GraphPad) “Two-way ANOVA” followed by “Bonferroni post-tests.” The levels of significance were of **p < 0.01 and ***p < 0.001 compared to the wild-type strain. *p < 0.1. **(A,B)** Acetate (1%) carbon source and **(C,D)** EtOH (1%) carbon source. *p < 0.1.

Decreased glucose consumption for the single and double mutants led us to conclude that the deletion of *atfA-D* genes impacts carbon source utilization. In the presence of acetate 1% as single carbon source, he Δ*atfA* Δ*atfB*, Δ*atfA* Δ*atfC*, and Δ*atfA* Δ*atfD* mimick the Δ*atfA* 50% growth reduction on acetate (Figures 5A,B, Table 1) while Δ*atfC*, Δ*atfD*, and Δ*atfC* Δ*atfD* have about 40% increased growth on acetate compared to the wild-type strain (Figures 5A,B, Table 1). In the presence of ethanol 1% as single carbon source, the Δ*atfC*, Δ*atfD*, Δ*atfB* Δ*atfC*, Δ*atfC* Δ*atfD* mutants have about 40% growth reduction when compared to the wild-type strain (Figures 5C,D, Table 1). There is an additive interaction in the Δ*atfA* Δ*atfB* double mutant (Figures 5C,D, Table 1).

Taken together, these data suggest a multi-factorial interaction between TFs AtfA-D that contributes to modulate metabolism allowing the adaptation of the fungus to stressful conditions, therefore impacting *A. fumigatus* ability to use different carbon sources.

### Accumulation of Trehalose and Glycogen Is Reduced in the Double Deletion Mutants

**Figure 6:**
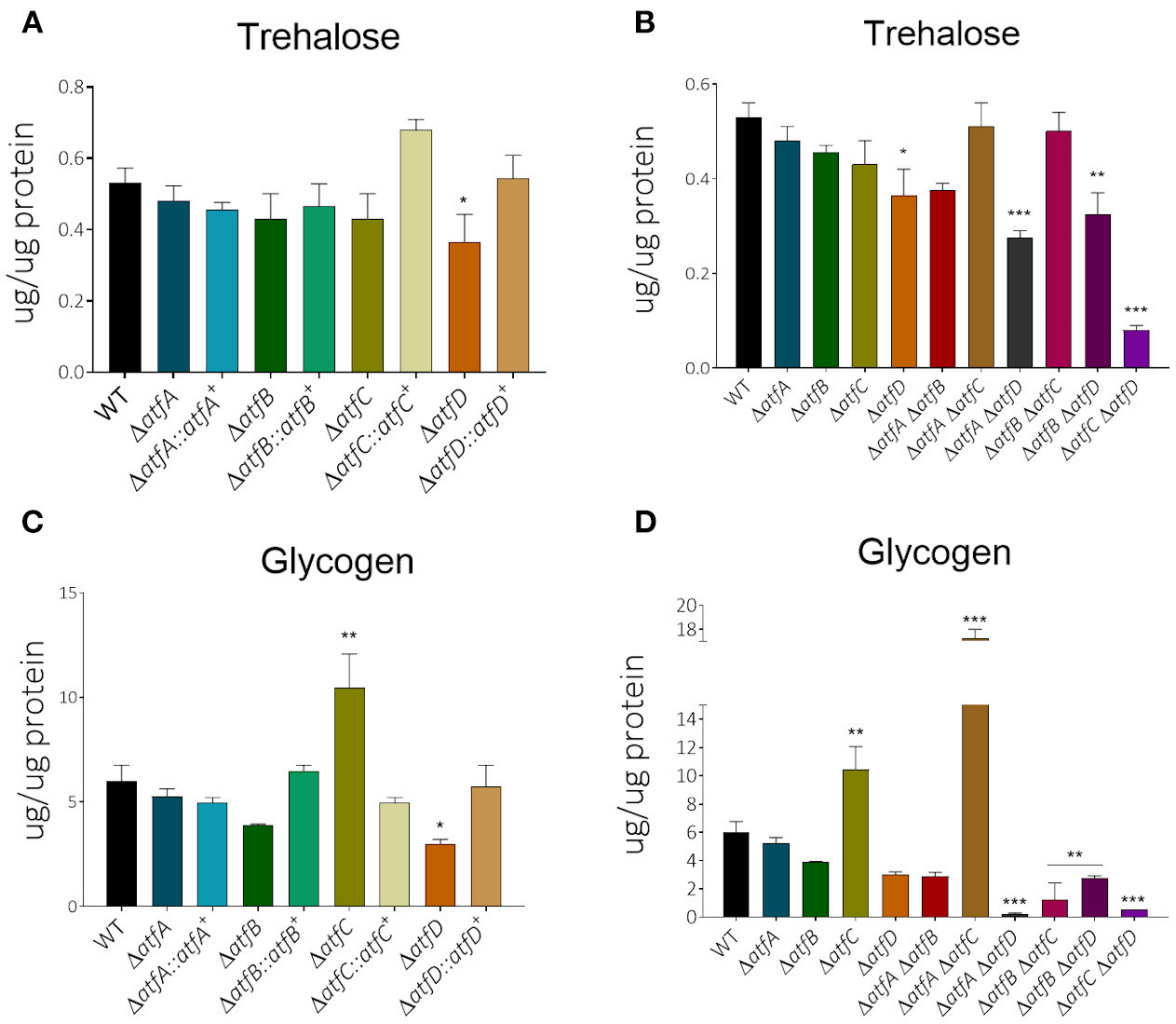
There is altered trehalose and glycogen accumulation in the Δ*atfA-D* single and double null mutants. The strains were grown for 24 or 48 h in MM+1% glucose. The results are the means of 3 repetitions for each strain ± standard deviation. The data were analyzed using (Prism, GraphPad) “Two-way ANOVA” followed by “Bonferroni post-tests.” The levels of significance were of *p < 0.1, **p < 0.01, and ***p < 0.001 compared to the wild-type strain. **(A,B)** Trehalose and **(C,D)** glycogen accumulation.

We have previously shown that MAPKs SakA and MpkC physically interact with protein kinase A and these protein kinases are necessary for normal accumulation/degradation of trehalose and glycogen (de Assis et al., 2018). The Δ*atfD*, Δ*atfA* Δ*atfD*, Δ*atfB* Δ*atfD*, and Δ*atfC* Δ*atfD* have about 30–50% reduction in trehalose accumulation when compared to the wild-type strain (Figures 6A,B, Table 1). There is about a 2-fold increased glycogen accumulation in Δ*atfD* and Δ*atfA* Δ*atfC* when compared to the wild-type (Figures 6C,D, Table 1). Interestingly, all the double mutants, except Δ*atfA* Δ*atfC*, have about 50–90% glycogen reduction when compared to the wild-type (Figures 6C,D, Table 1).

These data suggest that AtfA-D are important to modulate the metabolism of storage sugars, like trehalose and glycogen.

### The Genetic Interaction Between the atfA-D Genes Contributes to Virulence in *A. fumigatus*

**Figure 7:**
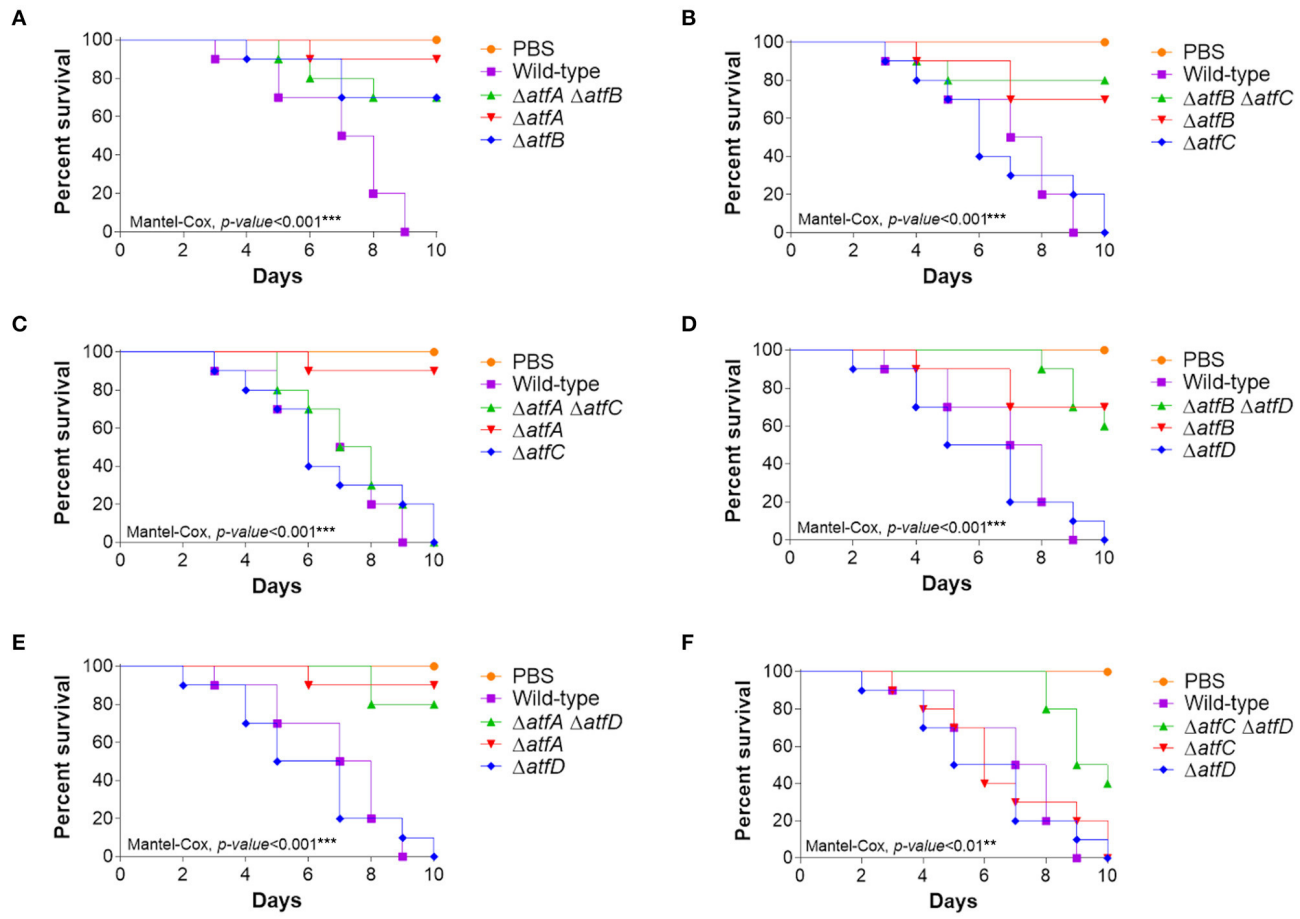
Virulence of *A. fumigatus* Δ*atfA-D* single and double null mutants in *Galleria mellonella*. **(A–F)** Cumulative survival in the model moth *Galleria mellonella*. Larvae infection was carried out via inoculation of 10^6^ conidia. For inoculations, 10 larvae were infected per wild-type and null mutant strains. The levels of significance were of *p < 0.1, **p < 0.01, and ***p < 0.001 compared to the wild-type strain. Phosphate buffered saline (PBS) was used as a negative control.

The single mutants Δ*atfA* and Δ*atfB* are either attenuated or avirulent in both *Galleria mellonella* larval model and in a neutropenic murine model (Silva et al., 2017). To evaluate the virulence of double deletion mutants we used the *G. mellonella* model of infection (Silva et al., 2017). The double mutant Δ*atfA* Δ*atfB* is avirulent and are epistatic to Δ*atfA* and Δ*atfB* (Figure 7A, Table 1) while *atfA* or *atfB* deleted in the Δ*atfC* or Δ*atfD* mimicked the avirulent phenotypes of Δ*atfA* and Δ*atfB* (Figures 7B–E, Table 1). Interestingly Δ*atfC* Δ*atfD* showed to be avirulent while Δ*atfC* suppressed the avirulent phenotype of Δ*atfA* (Figure 7F, Table 1).

These results suggest that AtfA-D interactions affect the *A. fumigatus* virulence in *G. mellonella*.

### SakA and TFs AtfA-D Physically Interact

**Figure 8:**
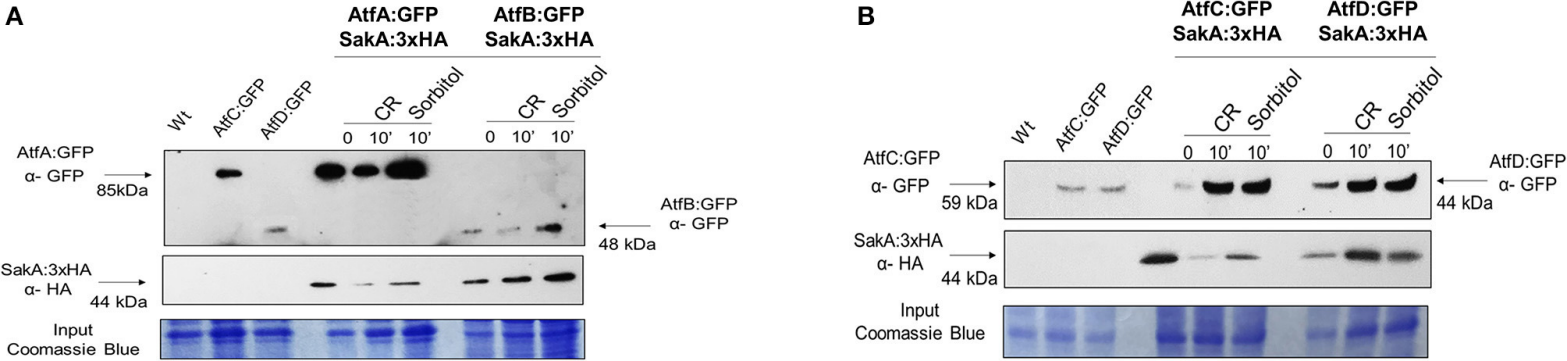
Co-Immunoprecipitation (Co-IP) assays between SakA and AtfA-D. **(A)** Verification of the association between AtfA:GFP and SakA:3xHA and association between AtfB:GFP and SakA:3xHA. **(B)** Verification of the association between AtfC:GFP and SakA:3xHA and association between AtfD:GFP and SakA:3xHA. Affinity purification assays from GFP-tagged AtfA-D strain in the background of 3xHA-tagged SakA were performed with GFP-Trap and anti-HA to verify interactions upon sorbitol and Congo Red (CR). Coimmunoprecipitated proteins were analyzed for the indicated antibodies. input staining with Coomassie blue.

Transcriptional analysis of *A. fumigatus* identified *atfA-D* mRNA accumulation as dependent on SakA and MpkC (Silva et al., 2017). *A. nidulans* AtfA physically interacts with SakA upon oxidative stress (Lara-Rojas et al., 2011). *A. fumigatus* SakA:3xHA physically interacts not only with AtfA:GFP, but also with AtfB-D:GFP in the absence and presence of osmotic (10 min exposure to 1.2 M sorbitol) and cell wall (10 min exposure to congo red 300 μg/ml) stresses (Figures 8A,B, Supplementary Figure 3).

These results suggest that SakA is directly or indirectly interacting with AtfA-D raising the hypothesis that SakA is directly or indirectly involved in the activation of TFs AtfA-D in *A. fumigatus*.

### Co-IP Reveals Interactions Between the *A. fumigatus* AtfA-D

**Figure 9:**
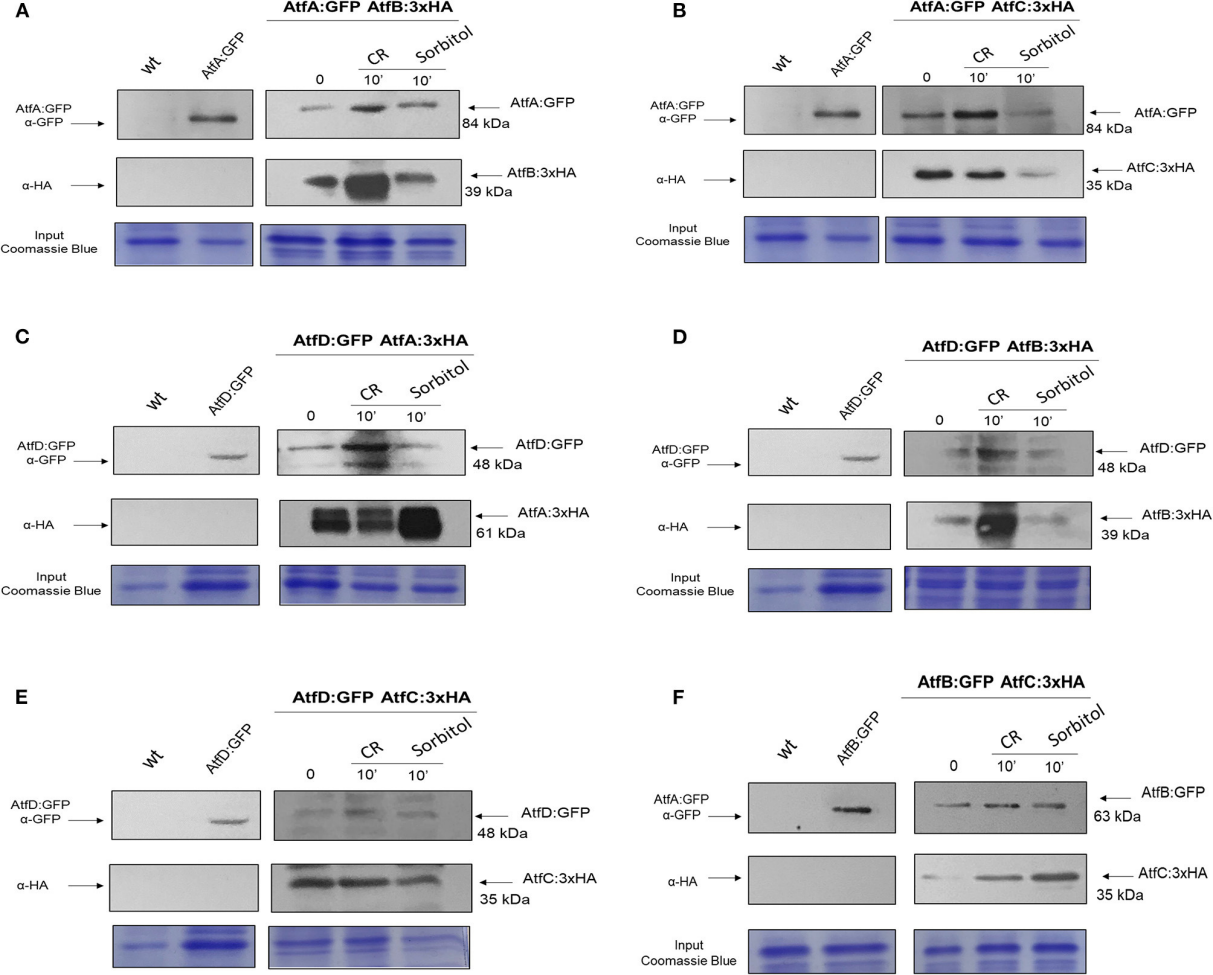
Co-Immunoprecipitation (Co-IP) interaction experiments between AtfA-D TFs. **(A)** Association between AtfA:GFP and AtfB:3xHA proteins. **(B)** Association between AtfA:GFP and atfC:3xHA proteins. **(C)** Association between AtfD:GFP and AtfA:3xHA proteins. **(D)** Association between AtfB:GFP and AtfC:3xHA proteins. **(E)** Association between AtfD:GFP and AtfB:3xHA proteins. **(F)** Association between AtfD:GFP and AtfC:3xHA. Affinity purification assays from GFP-tagged AtfA:GFP; AtfB:GFP and AtfD:GFP strain in the background of AtfA-, B-, and C:3xHA-tagged were performed with GFP-Trap and anti-HA to verify interactions upon sorbitol and Congo Red (CR). Coimmunoprecipitated proteins were analyzed for the indicated antibodies. input staining with Coomassie blue.

We constructed six different combinations of functional strains expressing AtfA-D:GFP and AtfA-D:3xHA (AtfA:GFP AtfB:3xHA, AtfA:GFP AtfC:3xHA, AtfD:GFP AtfA:3xHA, AtfB:GFP AtfC:3xHA, AtfD:GFP AtfB:3xHA, and AtfD:GFP AtfC:3xHA; Figure 9, Supplementary Figure 4). Subsequently, we evaluated through co-IP experiments the hypothesis that AtfA-D could physically interact forming heterodimers during different stressing conditions. AtfA:GFP interacted with AtfB, AtfC, and AtfD, both at before (T0) and after the addition of sorbitol or CR (Figures 9A–C). The TFs AtfC and AtfD also showed physical interaction in all three conditions (Figure 9E). In addition, AtfB interacted with AtfC in the three conditions T0, sorbitol, and CR (Figure 9F). The additional bands observed for AtfD:GFP and AtfA:3xHA (Figure 9C) and AtfD:GFP (Figure 9D) could be GFP degradation products and/or AtfA/AtfD intermediary phosphorylated forms.

Together, these data strongly suggest that the interactions of TFs are of great importance for efficient gene transcription, allowing the fungus to quickly and robustly respond to different growth conditions.

## Discussion

The exposure of fungal cells to different stressing conditions leads to the activation of MAPK cascades which are involved in maintenance and modulation of the adaptative response, resulting in increased resistance and ensuring the survival of the fungus. Signaling through MAPK cascades results in altered gene expression that regulates many processes, including pheromone response, filamentous growth, cell wall component biosynthesis, establishment of virulence, and mediation of drug resistance (Bahn et al., 2005; Valiante et al., 2008, 2009; de Castro et al., 2014). The *A. fumigatus* HOG pathway encompasses a MAPK cascade activated by phosphorylation and is triggered by stresses such as osmotic, oxidative, and damage to the cell wall, eventually resulting in the activation of the effector kinases SakA^Hog1p^ and MpkC (Bruder Nascimento et al., 2016). These protein kinases can then migrate to the nucleus and regulate the activity of additional protein targets, thus modulating a cellular response to the extracellular environment (Bruder Nascimento et al., 2016). Our group previously showed that in *A. fumigatus* the expression of the TFs AtfA and AtfB during osmotic stress was dependent on SakA^Hog1p^, while AtfC and AtfD were dependent on both MpkC and SakA^Hog1p^. Furthermore, *atfA-D* null mutants are more sensitive to oxidative and cell wall stresses (Supplementary Figures 3, 4 in Silva et al., 2017). In the present work, we studied possible genetic and protein interactions between AtfA-D. Our results indicate that there are several levels of genetic interactions among these TF mutants. However, *atfA* and *atfB* null mutants displayed epistasis in most of the stressing conditions here investigated. The single mutants Δ*atfA* and Δ*atfB* showed reduced virulence in *G. mellonella* while Δ*atfC* and Δ*atfD* were as virulent as the wild-type. However, genetic interactions only between Δ*atfC* and Δ*atfD* have decreased virulence in *G. mellonella* since the other double mutants are impacted by either Δ*atfA* or Δ*atfB*. Some AtfA-D homologs have already been characterized in other *Aspergilli*. In *A. niger*, AtfC homolog (An12g10230) has been shown to be involved in the formation of conidia and resistance to stress (van Leeuwen et al., 2013). In addition, the *A. niger atfC* showed high levels of transcription in dormant conidia and strong negative regulation during germination (van Leeuwen et al., 2013). The AtfB homolog in the *A. oryzae* is involved in many cellular processes, such as stress tolerance and amino acid metabolism (Sakamoto et al., 2008). In *A. parasiticus*, AtfB is a master regulator of three functional networks, secondary metabolism, stress response and conidiospore development (Wee et al., 2017). When *A. nidulans atfB* and *atfC* deletion mutants were tested for oxidative stress sensitivity, only the Δ*atfB* mutant was sensitive to H_2_O_2_ but not to *t*-butylhydroperoxide (Lara-Rojas et al., 2011). Interestingly, AtfA but not AtfB or AtfC is required for catalase A expression in *A. nidulans* conidia, clearly indicating a differential function for these transcription factors (Lara-Rojas et al., 2011). In *A. nidulans*, AtfB homolog is also related to conidia production since the deletion of *atfB* generated a strain whose conidia numbers were slightly reduced when compared to the wild-type (Sakamoto et al., 2008). These data indicate the involvement of AtfA, AtfB and AtfC during adaptation to osmotic and cell wall stress and in the production of conidia. In *A. fumigatus*, the reduction of conidiation was not observed for strains with single deletions or for strains with double deletions.

In *S. cerevisiae*, Hog1 kinase converts the Sko1-Cyc8-Tup1 repressor complex into an activator in response to osmotic stress (Proft and Struhl, 2002). *S. cerevisiae* Sko1p is a bZIP TF of the ATF/CREB family that targets ~40 genes with different biological functions, including response to hyperosmotic transcriptional stress (Rep et al., 2001; Proft et al., 2005). In *Schizosaccharomyces pombe*, activation of the MAPK pathway leads to phosphorylation of the MAPK HOG Spc1/Sty1^Hog1p^, which then accumulates in the nuclei, promoting activation and transcriptional repression of genes partially or completely dependent on *A. fumigatus* AtfA homolog Atf1p (Millar et al., 1995; Shiozaki and Russell, 1995; Wilkinson et al., 1996; Kawasaki et al., 2002). *S. pombe* Atf1 is important for the response to various stressing conditions and has the ability to form a heterodimer with another bZip TF, Pcr1p (Lawrence et al., 2007; Salat-Canela et al., 2017), which then participates in meiotic recombination, maintenance of the heterochromatin structure and gene regulation (Salat-Canela et al., 2017). Upon oxidative stress *A. nidulans* SakA is translocated to the nucleus and also physically interacts with AtfA, whose location is constitutively nuclear (Lara-Rojas et al., 2011; Kato et al., 2013; Salat-Canela et al., 2017).

In mammalian cells, the formation of heterodimers by TFs bZip type are suggested to control a large number of regulatory transcriptional systems, increasing the diversity of responses and the regulatory potential determined by TFs (Wolberger, 1998; Rodríguez-Martínez et al., 2017). Surprisingly, we observed a complex interaction among all four AtfA-D forming heterodimers and suggesting this possibility in *A. fumigatus*. In addition, we have demonstrated that *A. fumigatus* SakA physically interacts not only with AtfA but also with AtfB, -C, and -D. The TF AtfA^Atf1p^ of *A. fumigatus* interacts with sakA^Hog1p^ upon different stress conditions. In addition, we observed association among AtfB, AtfC, and AtfD with SakA^Hog1p^. These data indicate that these four TFs interact directly or indirectly physically with the SakA^Hog1p^ during transcriptional activation. It remains to be determined if their activation is dependent on SakA^Hog1p^. In *A. fumigatus*, AtfA is also constitutively present in the nuclei, regardless of the stressful conditions to which the fungus is exposed. In contrast, only a fraction (~10%) of AtfB-D:GFP is located in the nuclei. However, after exposure to osmotic, oxidative or cell wall stresses, migration of AtfB, -C, and -D to the nucleus occurs in ~80%. These results indicate that the mechanism of activation of AtfB-D is through initial translocation to the nucleus while AtfA is already present in the nucleus. All these proteins could be possibly functionally activated by phosphorylation by their interaction with SakA. It remains to be investigated if SakA directly phosphorylates AtfA-D and what are the AtfA-D targets that are modulated during these stressing conditions.

We observed the single and double Δ*atfA-D* mutants are more susceptible to cell wall damaging agents, suggesting an altered cell wall composition. *A. fumigatus* is capable of using different carbon sources and this characteristic is important for its survival within the host and, therefore, for virulence. The single (although minor) and double mutants *atfA-D* mutants showed a reduction in glucose uptake and growth in the presence of glucose as single carbon source. Interestingly, the mutant Δ*atfA* showed a reduction in its ability to metabolize acetate while Δ*atfC* and Δ*atfD* have increased growth in the presence of acetate as single carbon source compared to the wild-type strain. In contrast, Δ*atfC* and Δ*atfD* have decreased growth in the presence of glycerol as single carbon source.

Trehalose is an important source of energy for fungi, protecting cells, preventing the aggregation of denatured proteins, promoting the elimination of free radicals, and helping cells to resist to environmental stress and nutrient deprivation (Thammahong et al., 2017). Recent studies have shown that trehalose biosynthesis may be involved in mediating the response to stress and also in the virulence of pathogenic fungi (Martínez-Esparza et al., 2007). Our data showed that the single Δ*atfD* mutant and the double mutants Δ*atfA* Δ*atfD*, Δ*atfB* Δ*atfD*, and Δ*atfC* Δ*atfD* reduced trehalose accumulation when compared to the wild type, suggesting that AtfD is important for transcriptional regulation of genes involved in trehalose biosynthesis. The conidia of the mutant strain Δ*atfA*, have previously been shown to reduce trehalose accumulation (Hagiwara et al., 2014). However, our results revealed that Δ*atfA* has normal trehalose levels in mycelia, suggesting that AtfA is important only for trehalose accumulation in conidia. A possible explanation is provided by *A. nidulans* where AtfA is necessary for the presence of SakA in conidia, but not in the mycelia (Lara-Rojas et al., 2011). The glycogen levels were also quantified and single mutants Δ*atfC* and Δ*atfD* have increased and decreased glycogen levels, respectively, when compared to the wild-type strain. Interestingly, there is a synergistic effect between Δ*atfA* and Δ*atfB* since in the double mutant there is decreased glycogen accumulation.

These results strongly suggest that AtfA-D are able to modulate genes involved in the primary metabolism, most likely genes related to carbon source assimilation. It remains to be investigated the role played by SakA and MpkC in this process and if this effect on primary carbon metabolism is indirectly affecting trehalose and glycogen accumulation, and/or if the genes involved in the metabolism of these storage sugars are directly regulated by AtfA-D.

The MAPK-HOG SakA and MpkC pathway is important for maintaining intracellular glycogen and trehalose levels, which are necessary for glucose uptake and intracellular glycemic signaling (de Assis et al., 2015). These data corroborate the data obtained by our study, showing once again that SakA interacts with TFs AtfA-D during metabolic regulation. In addition, impaired glucose uptake and use for single and double AtfA-D mutants may result in altered cell wall composition, which would explain the sensitivity of the mutants to different cell wall stressors. Protein kinase A (PKA) pathway regulation is important for the use of carbon sources and the degradation of trehalose and glycogen (Freitas et al., 2010). Therefore, our studies suggest that AtfA-D could participate in this regulatory cascade, activating or repressing important targets for carbon source assimilation and sugar storage.

## Data Availability Statement

The original contributions presented in the study are included in the article/Supplementary Material, further inquiries can be directed to the corresponding author/s.

## Author Contributions

LS realized formal analysis, investigation, and wrote original draft, review, and editing. MH wrote, review, and editing. GG conceptualization, funding acquisition, project administration, supervisioned, and wrote original draft, review, and editing the manuscript. All authors revised the manuscript.

## Conflict of Interest

The authors declare that the research was conducted in the absence of any commercial or financial relationships that could be construed as a potential conflict of interest.
