# Supplementary material for: Genetic Interactions Between Aspergillus fumigatus Basic Leucine Zipper (bZIP) Transcription Factors AtfA, AtfB, AtfC, and AtfD
Source: Front Fungal Biol. 2021 Feb 11;2:632048. doi: 10.3389/ffunb.2021.632048 (PMC10512269; doi:10.3389/ffunb.2021.632048)
Supplement: Supplementary file 1 [file Presentation_1.PPTX]

## Slide 1
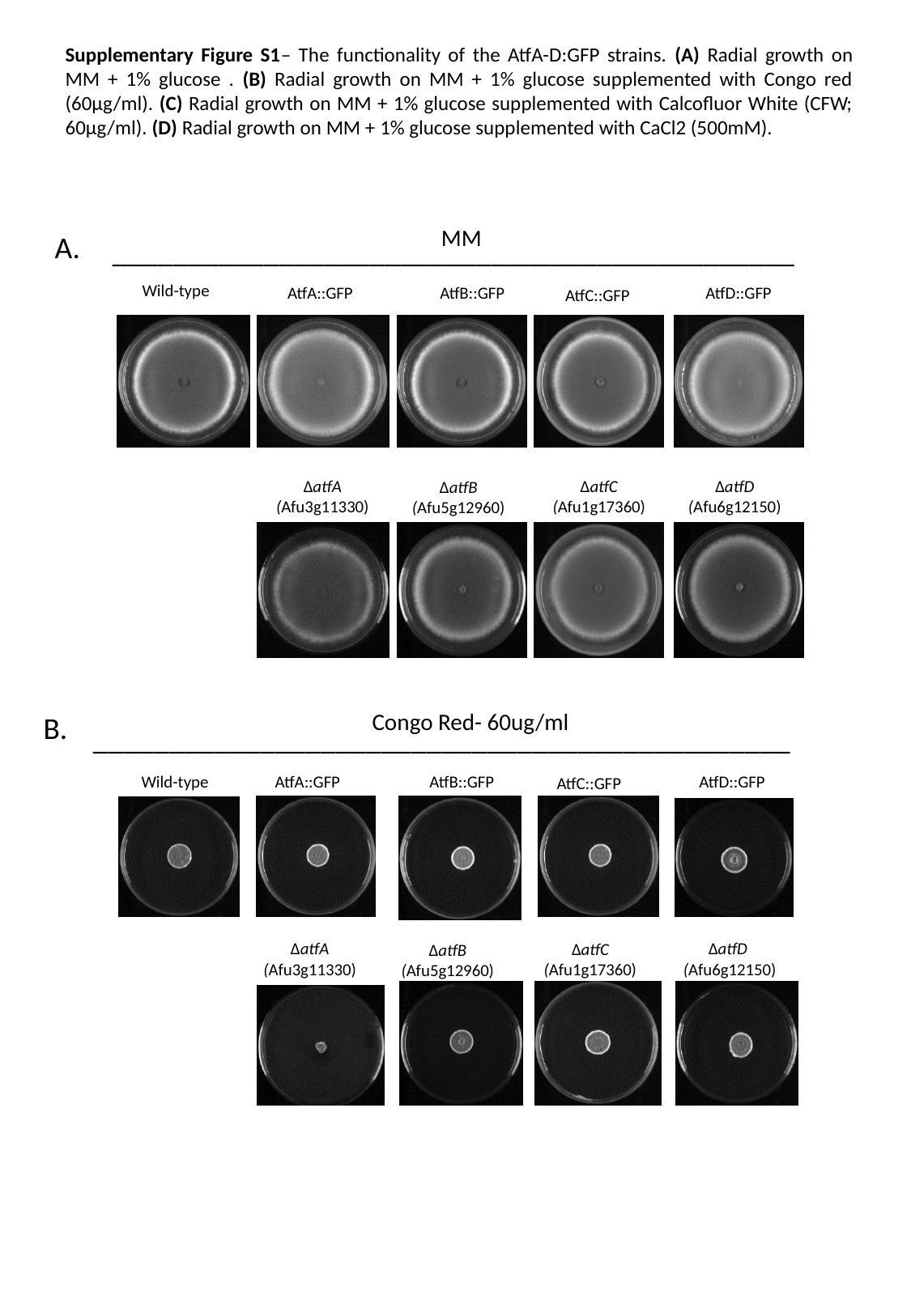

Supplementary Figure S1– The functionality of the AtfA-D:GFP strains. (A) Radial growth on MM + 1% glucose . (B) Radial growth on MM + 1% glucose supplemented with Congo red (60µg/ml). (C) Radial growth on MM + 1% glucose supplemented with Calcofluor White (CFW; 60µg/ml). (D) Radial growth on MM + 1% glucose supplemented with CaCl2 (500mM).
MM
A.
_____________________________________________
Wild-type
AtfA::GFP
AtfB::GFP
AtfD::GFP
AtfC::GFP
∆atfA
(Afu3g11330)
∆atfD
(Afu6g12150)
∆atfC
(Afu1g17360)
∆atfB
(Afu5g12960)
Congo Red- 60ug/ml
B.
______________________________________________
Wild-type
AtfA::GFP
AtfB::GFP
AtfD::GFP
AtfC::GFP
∆atfA
(Afu3g11330)
∆atfD
 (Afu6g12150)
∆atfC
(Afu1g17360)
∆atfB
(Afu5g12960)

## Slide 2
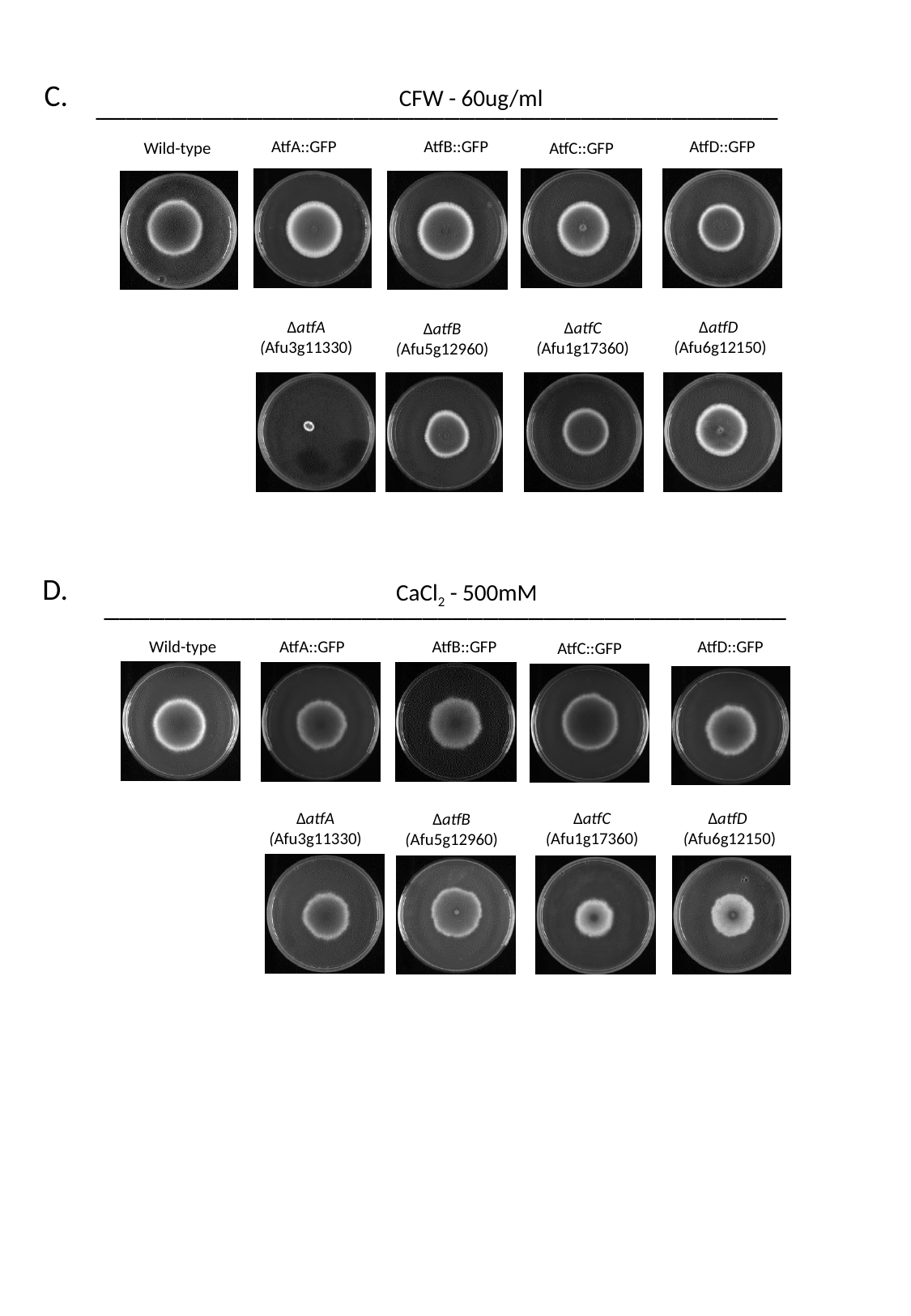

C.
CFW - 60ug/ml
_____________________________________________
AtfA::GFP
AtfB::GFP
AtfD::GFP
Wild-type
AtfC::GFP
∆atfA
(Afu3g11330)
∆atfD
 (Afu6g12150)
∆atfC
(Afu1g17360)
∆atfB
(Afu5g12960)
D.
CaCl2 - 500mM
_____________________________________________
Wild-type
AtfA::GFP
AtfB::GFP
AtfD::GFP
AtfC::GFP
∆atfA
(Afu3g11330)
∆atfD
 (Afu6g12150)
∆atfC
(Afu1g17360)
∆atfB
(Afu5g12960)
