# Supplementary material for: Genetic Interactions Between Aspergillus fumigatus Basic Leucine Zipper (bZIP) Transcription Factors AtfA, AtfB, AtfC, and AtfD
Source: Front Fungal Biol. 2021 Feb 11;2:632048. doi: 10.3389/ffunb.2021.632048 (PMC10512269; doi:10.3389/ffunb.2021.632048)
Supplement: Supplementary file 2 [file Presentation_2.PPTX]

## Slide 1
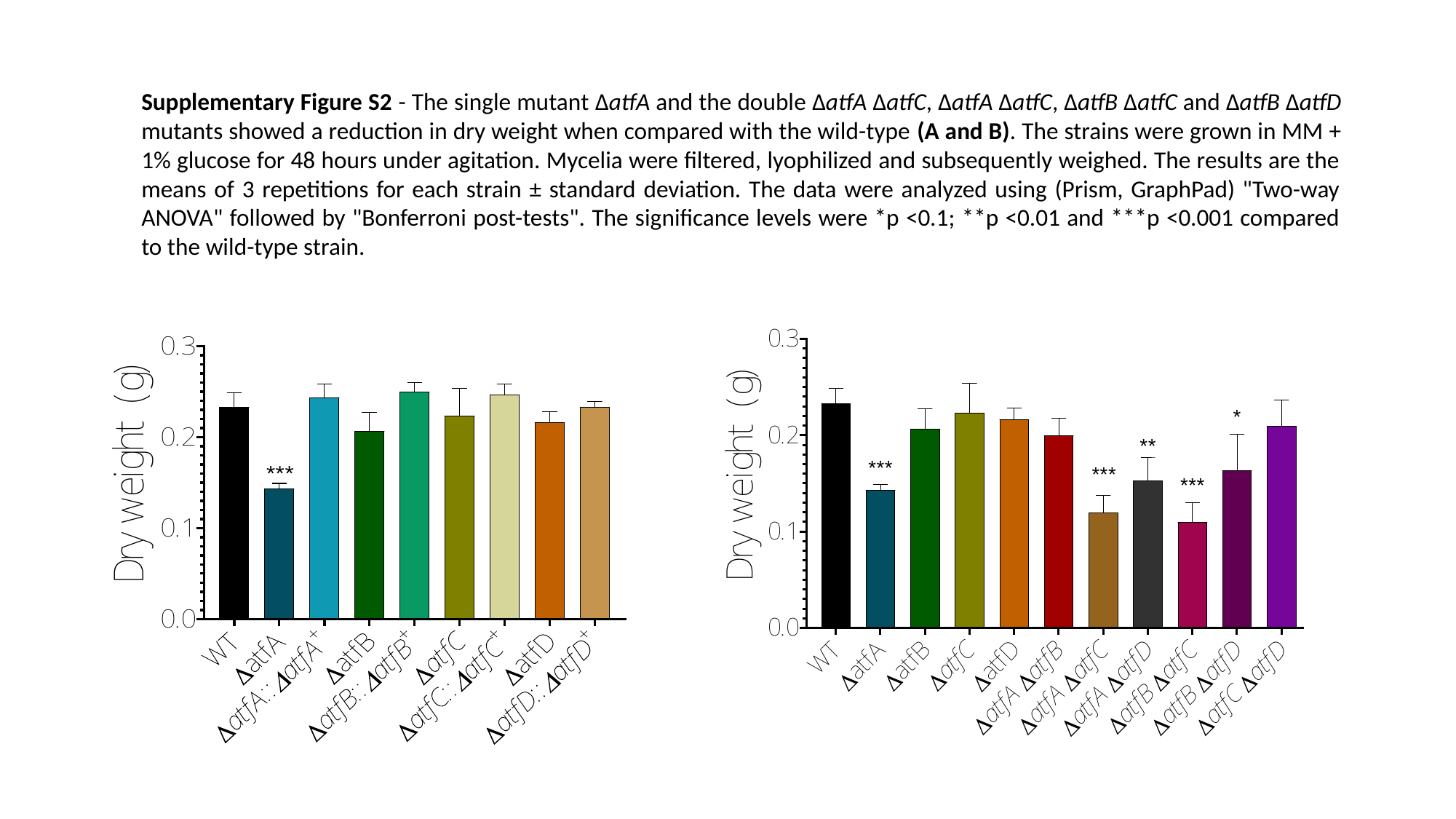

Supplementary Figure S2 - The single mutant ΔatfA and the double ΔatfA ΔatfC, ΔatfA ΔatfC, ΔatfB ΔatfC and ΔatfB ΔatfD mutants showed a reduction in dry weight when compared with the wild-type (A and B). The strains were grown in MM + 1% glucose for 48 hours under agitation. Mycelia were filtered, lyophilized and subsequently weighed. The results are the means of 3 repetitions for each strain ± standard deviation. The data were analyzed using (Prism, GraphPad) "Two-way ANOVA" followed by "Bonferroni post-tests". The significance levels were *p <0.1; **p <0.01 and ***p <0.001 compared to the wild-type strain.
