# Supplementary material for: Genetic Interactions Between Aspergillus fumigatus Basic Leucine Zipper (bZIP) Transcription Factors AtfA, AtfB, AtfC, and AtfD
Source: Front Fungal Biol. 2021 Feb 11;2:632048. doi: 10.3389/ffunb.2021.632048 (PMC10512269; doi:10.3389/ffunb.2021.632048)
Supplement: Supplementary file 3 [file Presentation_3.PPTX]

## Slide 1
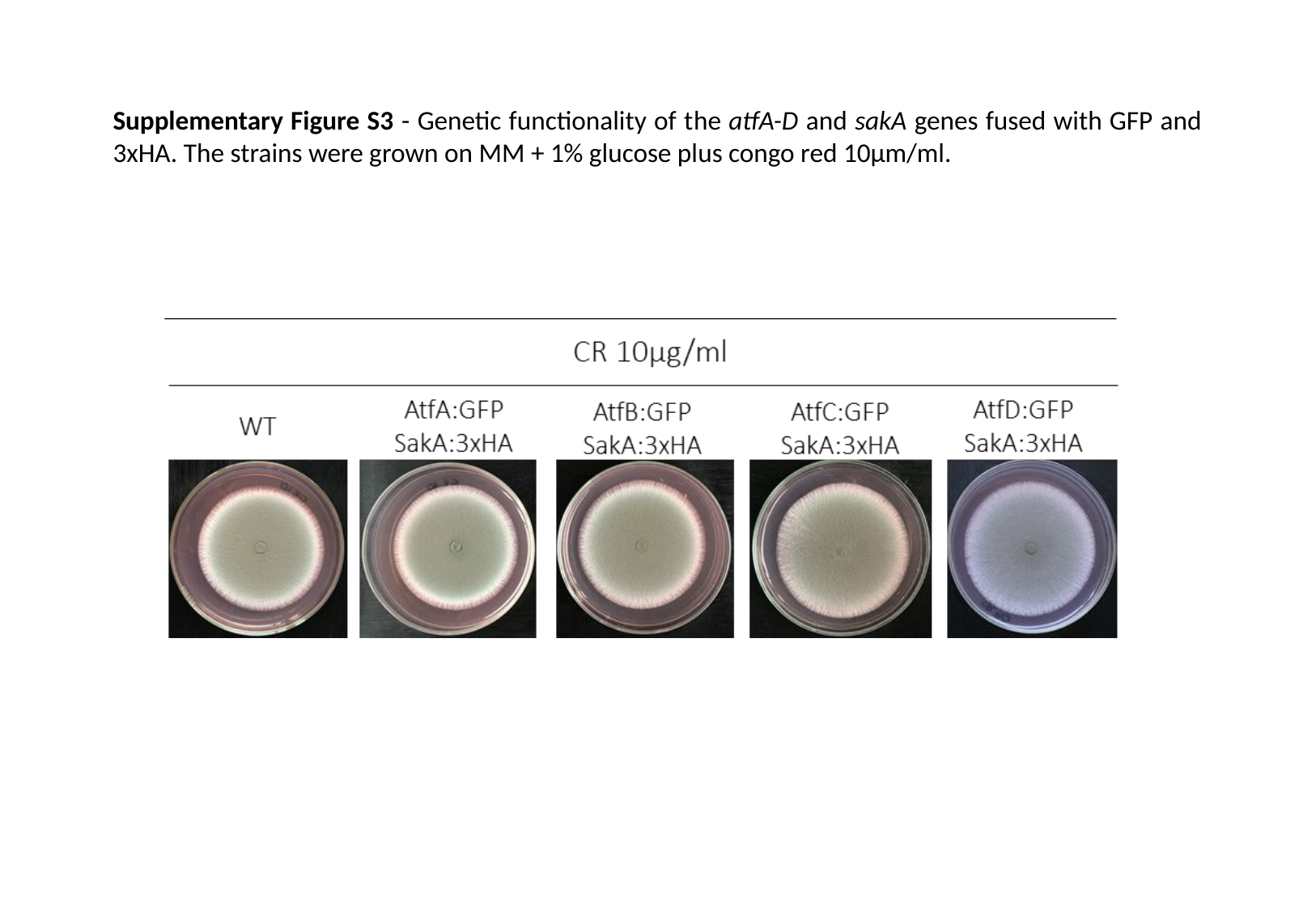

Supplementary Figure S3 - Genetic functionality of the atfA-D and sakA genes fused with GFP and 3xHA. The strains were grown on MM + 1% glucose plus congo red 10µm/ml.
